# Dr. Alban S. Payne

**Published:** 1872-10

**Authors:** 


### DR. ALBAN S. PAYNE,

Favors us with another of his practical articles, this time upon "Eclampsia." The Dr. is a 'live man,' and an honor to his profession. We hope to hear from him often.

